# Mathematical Modeling of HIV Prevention Measures Including Pre-Exposure Prophylaxis on HIV Incidence in South Korea

**DOI:** 10.1371/journal.pone.0090080

**Published:** 2014-03-24

**Authors:** Sun Bean Kim, Myoungho Yoon, Nam Su Ku, Min Hyung Kim, Je Eun Song, Jin Young Ahn, Su Jin Jeong, Changsoo Kim, Hee-Dae Kwon, Jeehyun Lee, Davey M. Smith, Jun Yong Choi

**Affiliations:** 1 Division of Infectious Disease, Department of Internal Medicine, Severance Hospital, Yonsei University College of Medicine, Seoul, South Korea; 2 AIDS Research Institute, Severance Hospital, Yonsei University College of Medicine, Seoul, South Korea; 3 Department of Mathematics, Yonsei University, Seoul, South Korea; 4 Department of Preventive Medicine, Yonsei University College of Medicine, Seoul, South Korea; 5 Department of Mathematics, Inha University, Incheon, South Korea; 6 Department of Computational Science and Engineering, Yonsei University, Seoul, South Korea; 7 Department of Medicine, University of California San Diego, La Jolla, California, United States of America; 8 Veterans Affairs San Diego Healthcare System, San Diego, California, United States of America; Alberta Provincial Laboratory for Public Health/University of Alberta, Canada

## Abstract

**Background:**

Multiple prevention measures have the possibility of impacting HIV incidence in South Korea, including early diagnosis, early treatment, and pre-exposure prophylaxis (PrEP). We investigated how each of these interventions could impact the local HIV epidemic, especially among men who have sex with men (MSM), who have become the major risk group in South Korea. A mathematical model was used to estimate the effects of each these interventions on the HIV epidemic in South Korea over the next 40 years, as compared to the current situation.

**Methods:**

We constructed a mathematical model of HIV infection among MSM in South Korea, dividing the MSM population into seven groups, and simulated the effects of early antiretroviral therapy (ART), early diagnosis, PrEP, and combination interventions on the incidence and prevalence of HIV infection, as compared to the current situation that would be expected without any new prevention measures.

**Results:**

Overall, the model suggested that the most effective prevention measure would be PrEP. Even though PrEP effectiveness could be lessened by increased unsafe sex behavior, PrEP use was still more beneficial than the current situation. In the model, early diagnosis of HIV infection was also effectively decreased HIV incidence. However, early ART did not show considerable effectiveness. As expected, it would be most effective if all interventions (PrEP, early diagnosis and early treatment) were implemented together.

**Conclusions:**

This model suggests that PrEP and early diagnosis could be a very effective way to reduce HIV incidence in South Korea among MSM.

## Introduction

The HIV epidemic has continued unabated in South Korea since 1985 with 700–800 new infections every year.[1] The epidemiology has changed over this time with a gradual shift from transmissions from heterosexual to male homosexual exposures being the greatest risk.[1], [2] As a high-income country with a low HIV prevalence (<0.03%) and the predominant transmission by men who have sex with men (MSM), the implementation of proper preventive measures using resources is important to minimize the HIV epidemic. However, given the current lack of change in HIV incidence but changing epidemiology, it is unclear what preventive measures should be adopted to thwart the epidemic. Mathematical models can be useful in helping guide public health interventions or planning for future clinical research trials, including HIV treatment. So far, some studies concerning the effect of antiretroviral treatment (ART) on HIV incidence using the mathematical analysis have been reported.[3]–[5] For example, the possible effects of the widespread use of ART and levels of unsafe sex on HIV incidence among MSM in San Francisco and Sydney have been evaluated using mathematical models.[4], [5] Both of these models have informed public health strategies and are relevant to the model developed in this study.

The introduction of ART into clinical practice has led to dramatic reductions in morbidity and in mortality associated with HIV infection.[6]–[8] Several studies have also observed the beneficial effects of ART for HIV-infected individuals on the sexual transmission of the virus to their sexual partners.[9], [10] For HIV-uninfected high-risk individuals, ART has also been effective in the prevention of HIV acquisition (iPrEX Trial).[10] Based on the results of iPrEx study, the United States Centers for Disease Control and Prevention (CDC) has developed guidelines on the use of tenofovir and emcitritabine as pre-exposure prophylaxis (PrEP) for MSM at high risk for HIV acquisition in the United States.[11] However, it is not clear how the implementation of PrEP impacts the incidence of HIV infection in "real life", non-trial settings. Concerns have been raised as to whether PrEP could result in increased risky sexual practices through behavioral disinhibition. It is possible that individuals may feel biologically protected and decide to abandon condom use, underscoring the importance of implementing behavioral modification and encouraging continued condom use if PrEP is to be a successful public health intervention.[12] In this study, using mathematical model, we estimate the effects of a variety of prevention interventions, including PrEP, on the HIV incidence and prevalence among MSM in South Korea based on best available data and compared to the current situation and trends.

## Methods

### The model and the parameters

**Figure 1:**
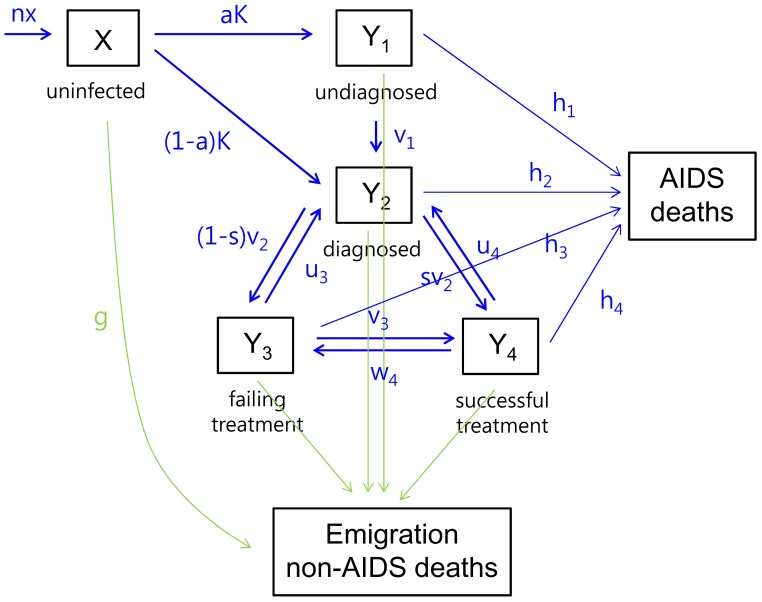
Multistate HIV infection model for a MSM population. NOTE: See table 1 and S1 for referring the meaning of each abbreviations

We constructed a mathematical model of HIV infection among MSM population in South Korea (Fig. 1 and Fig. S1). The model aimed to evaluate the effect of preventive interventions on the time trends of the incidence and prevalence of HIV infection in MSM population of South Korea. The model divided the MSM population into seven groups: uninfected MSM (X); HIV-infected MSM undiagnosed (Y_1_); HIV-infected MSM diagnosed without treatment (Y_2_); HIV-infected MSM with ART failure (Y_3_); HIV-infected MSM with successful ART (Y_4_); HIV-infected MSM with AIDS-related mortality; and HIV-infected MSM who emigrated from the sexually active MSM community or have non-AIDS mortality.[13] Since the last two groups did not affect changes of MSM population in the other groups, our model included five differential equations describing the dynamics of the five groups (X, Y_1_, Y_2_, Y_3_, and Y_4_). The developed model was adapted from Clements et al,[3] where the compartments were subject to diagnosis and treatment. Since viral load is a critical factor for HIV transmission or infection rate, and ART can reduce and maintain low viral loads, we considered this in the model. Specifically, among the treated patients, 70% were considered to be successful in the suppression of viral loads and the rest were considered to have failed.[14] This is reflected in our model by splitting the treated group into two separate compartments of treatment failure (Y_3_) and successful treatment.(Y_4_) In addition, the PrEP efficacy parameter, *f_p_*, was included in the model because ART for non-HIV infected MSM group could reduce the incidence of HIV infection.

The current study is based on derivative values, which are distributed in ranges but are not numerical. Table 1 shows the derivative values for each of the variables, parameters and references (see Table S1 for more details). The values were decided by the best available local epidemiological data, literature reviews, investigator's derivations, and calculations based on mathematical formulas. Some parameters (number of uninfected MSM [X]; number of undiagnosed, infected MSM [Y_1_];number of diagnosed; infected MSM without treatment [Y_2_]; number of infected MSM with treatment failure [Y_3_]; number of infected MSM with successful treatment [Y_4_]; proportion of successful treatments [s];rate of decline for undiagnosed [h_1_];and rate of decline for diagnosed [h_2_]) were decided based on the best available local epidemiological data. The best available local epidemiological data described the authenticity as demonstrated by references cited in the text. Some parameters (the number of new uninfected homosexual men each year [nx]; diagnosis rate [v_1_]; treatment uptake rate [v_2_]; and rate of AIDS death for infected MSM after treatment failure[h_3_]) were derived from the previous studies.[4], [15]–[19] nx was decided as 2,100 to 4,500 persons, derived from the finding that the annual proportion of new uninfected MSM would be 3% among the whole MSM population (70,000 to 150,000 persons).[4] v_1_ was computed as 0.1667 for those not diagnosed at seroconversion, derived from the finding that new HIV-infected men would be diagnosed in 5∼7 years on average.[15], [16] v_2_ was computed as 1.4794, derived from the finding that 95% of HIV-diagnosed men would start ART in 2.025 years on average.[18] In terms of proportion of ART use among MSM, 95% ART use within one year of diagnosis was selected as a possible maximal value that could be achieved in the real world. Values for the other parameters were taken by referring to the previous studies shown in Table 1 and Table S1.[3], [14], [17], [20]–[26]

**Table 1:** Variables and parameters for model.

Variables/Parameters	Meaning	Value	Unit	References
X	number of uninfected MSM	133077	number	[25]
Y_1_	number of undiagnosed, infected MSM	3438	number	[25]
Y_2_	number of diagnosed, infected MSM without treatment	1213	number	[1]
Y_3_	number of infected MSM with treatment failure	1219	number	[14]
Y_4_	number of infected MSM with successful treatment	2843	number	[14]
nx	number of new uninfected MSM each year	3300	number/year	Derivation
a	proportion of new infections undiagnosed at seroconversion	0.5	dimensionless	[3]
v_1_	diagnosis rate	0.1667	1/year	Derivation
v_2_	treatment uptake rate	1.4794	1/year	Derivation
s	proportion of successful treatments	0.8816	dimensionless	[14]
u_3_	treatment cessation rate due to treatment failure	0.3	1/year	[20]
u_4_	treatment cessation rate due to successful treatment	0.15	1/year	[20]
v_3_	treatment success rate	0.3542	1/year	[21], [23]
w_4_	treatment relapse rate	0.235	1/year	[26]
h_1_	rate of AIDS death for undiagnosed and diagnosed MSM	1/10		[22]
h_2_	rate of AIDS death for undiagnosed and diagnosed MSM	1/10	1/year	[22]
h_3_	rate of AIDS death for infected MSM after treatment failure	1/17	1/year	Derivation
h_4_	rate of AIDS death for infected MSM after successful treatment	1/32	1/year	[17], [24]
g	background migration rate for MSM	1/33.3	1/year	[3]
bc	average probability of HIV transmission occurring to a partner (infectiousness) * average annual number of HIV-infected partners with whom uninfected men have unprotected anal intercourse	0.8	1/year	[4]
*f_u_*	increased level of UAIC	1.1	dimensionless	[3]
*f_d_*	effect of diagnosis on reducing the rate of UAIC between uninfected and diagnosed men	0.5	dimensionless	[3]
*f_tf_*	average decrease in infectiousness as a result of treatment failure	0.4	dimensionless	[9]
*f_ts_*	average decrease in infectiousness as a result of successful treatment	0.04	dimensionless	[9]
*f_p_*	average decrease in infectiousness as a result of PrEP	0.56	dimensionless	[10]
K	HIV infection rate for uninfected MSM	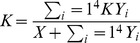	1/year	Calculation by transmission equations
K_1_	HIV infection rate for infected, undiagnosed MSM	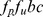	1/year	Calculation by transmission equations
K_2_	HIV infection rate for infected, diagnosed MSM	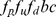	1/year	Calculation by transmission equations
K_3_	HIV infection rate for infected MSM with treatment failure	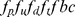	1/year	Calculation by transmission equations
K_4_	HIV infection rate for infected MSM with successful treatment	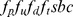	1/year	Calculation by transmission equations

**NOTE.** MSM, men who sex with men; UAIC, unprotected anal intercourse.

Value of variables (X, Y_1_, Y_2_, Y_3_, Y_4_) meant to be initial value for mathematical model. Value of parameters (from nx to *f_p_*) meant to be current value for model.

a, s, v_3_, and v_4_ are calculated by following equation: If there z% in t years, then the yearly rate r was computed by 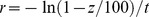.

v_1_, v_2_, h_1_, h_2_, h_3_, and h_4_ are calculated by following equation: If it takes t years on average to move to the next compartment, then the yearly rate r was computed by 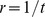.

K,K_1_,K_2_,K_3_,K_4_ are calculated by transmission equations supplemented by Fig. S1.

Dimensionless: it has no unit, such as a ratio or a percentage.

The preventive interventions of interests were: early diagnosis, early ART, and PrEP. Because some reports had concerns, such as interventions could affect the level of risky sexual behaviors, [27], [28] the model considered the relationship between the interventions and the level of unprotected anal intercourse. New HIV infections in uninfected MSM were modeled based on the average number of HIV-infected partners with whom the MSM would have unprotected anal intercourse (UAIC), and the average possibility of HIV transmission occurring with that partner (infectiousness). The investigator derived that the HIV diagnosis could reduce the level of UAIC between uninfected men and HIV-diagnosed men up to 50% (*f_d_* =  0.5), and the successful ART could reduce the infectiousness up to 96% (*f_ts_* = 0.04).[3] Referring to a previous study,[3] the proportion of new HIV infections undiagnosed at seroconversion (a) was derived as 50%. Through this procedure, the rest of parameters, including the HIV infection rate for uninfected MSM (K), undiagnosed (K_1_), diagnosed (K_2_), treatment failure (K_3_), and successful treatment (K_4_) were calculated by the transmission equations. The annual number of men within each group and the annual number of newly infected MSM (KX) were computed by simulating the mathematical model by assigning values to the parameters and initial states. (Fig. 1 and Fig. S1)

### Interventions scenarios

To mirror the uncertainty in many of factors included in our model, a simulation approach was introduced. We assumed five scenarios:

**Scenario 1:** 95% of HIV-diagnosed MSM take ART within 1 year of diagnosis.

**Scenario 2**: HIV-infected MSM know their disease within 1 year of infection.

**Scenario 3:** PrEP decreased HIV infectivity by 44%, while unsafe sex behavior did not increase.

**Scenario 4-1:** PreP decreased HIV infectivity by 44%, and unsafe sex behavior increased by 10%.

**Scenario 4-2:** PreP decreased HIV infectivity by 44%, and unsafe sex behavior increased by 20%.

**Scenario 4-3:** PreP decreased HIV infectivity by 44%, and unsafe sex behavior increased by 30%.

**Scenario 5:** Combined the following factors: HIV-infected MSM would be diagnosed within 1 year of infection, 99% of HIV-diagnosed MSM take ART within 1 year of diagnosis, and PrEP decreased HIV infectivity by 44% while unsafe sex behavior did not increase at all.

We estimated the effects of each these scenarios on the HIV incidence (KX) and prevalence (Y_1_ to Y_4_) over the next 40 years, as compared to the current situation which was defined as future epidemic that would be expected without any new prevention measures.

### Outcomes involving uncertainty

**Figure 2:**
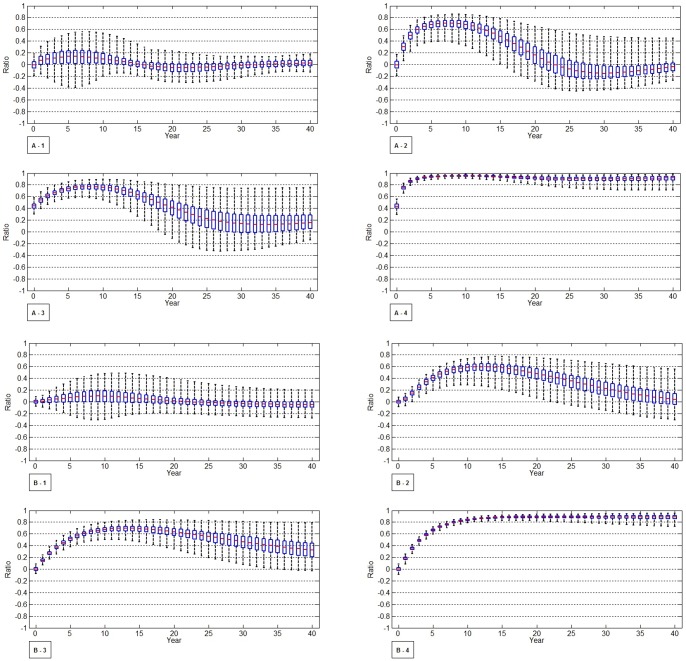
Modeled HIV incidence under different scenarios. A-1. Ratio of incident HIV cases (KX) compared early ART (scenario 1) with current situation. A-2. Ratio of incident HIV cases (KX) compared early diagnosis (scenario 2) with current situation. A-3. Ratio of incident HIV cases (KX) compared PrEP (scenario 3) with current situation. A-4. Ratio of incident HIV cases (KX) compared all interventions (scenario 5) with current situation. B-1. Ratio of prevalent HIV cases (Y_1–4_) compared early ART (scenario 1) with current situation B-2. Ratio of prevalent HIV cases (Y_1–4_) compared early diagnosis (scenario 2) with current situation. B-3. Ratio of prevalent HIV cases (Y_1–4_) compared PrEP (scenario 3) with current situation. B-4. Ratio of prevalent HIV cases (Y_1–4_) compared all interventions (scenario 5) with current situation. NOTE: The boxplot contains the median value of the data (horizontal red line), and extends from the first to the third quartile when simple random sampling with uniform distributions between ±10% of baseline of all parameters are used. Whisker bars are extended to minimum and maximum. Figures are the simulations of scenarios 1, 2, and 3, and 5. The X-axis represented the time sequence from initial to 40 years, and the Y-axis represented the ratio of HIV incidence (KX) and prevalence (Y_1–4_) compared with current status. Plots over zero represented a decrease over HIV incidence and prevalence than the current situation, while plots under zero meant an increase in HIV incidence and prevalence over current situation.

**Figure 3:**
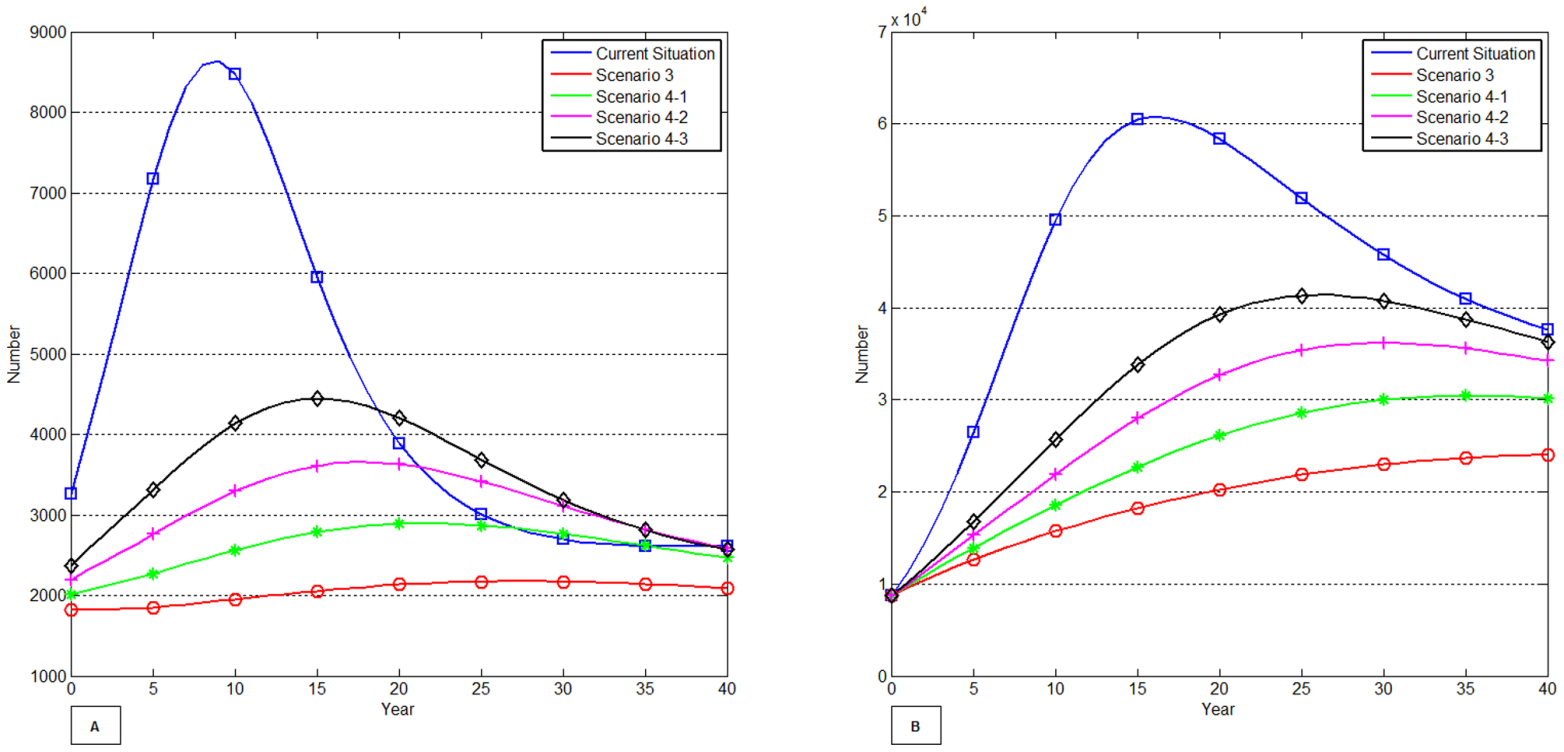
Comparison of number of incident HIV cases (KX) and prevalent HIV cases (Y_1–4_) between current situation and efficacy of PrEP modulated by unsafe sex practices (scenario 3 to 4-1,4-2, and 4-3). A. Comparison of number of incident HIV cases (KX) between current situation and scenarios 3 to 4-1, 4-2, and 4-3. B. Comparison of number of prevalent HIV cases (Y_1–4_) between current situation and efficacy of PrEP modulated by unsafe sex practices (scenario 3 to 4-1, 4-2, and 4-3). NOTE: Stream of current status, scenarios 3. 4-1, 4-2, and 4-3. Figure 3A: X-axis represented time sequence from now to 40 years and Y-axis represented stream of number of incident HIV cases (KX) compared to current situation with PrEP scenarios 3 to 4-1,4-2,4-3. Figure 3B: X-axis represented time sequence from now to 40 years, and Y-axis represented the number of prevalent HIV cases (Y_1_ to Y_4_) comparing current situation with PrEP scenarios 3, 4-1, 4-2, and 4-3.

Among the various outcomes, “KX” and “Y_1_ to Y_4_” were selected to evaluate the time trend of HIV incidence and prevalence, respectively. The number of newly infected MSM is meant to be KX. The number of current HIV infected MSM regardless of diagnosis or treatment is meant to be Y_1_ to Y_4_. The outcomes were examined up to 40 years after each intervention considering the increasing life expectancy of HIV-infected persons. To reflect the uncertainty of parameters, simple random sampling with uniform distributions between ± 10% of baseline of all parameters were assumed. In Fig. 2, 1000 simulations are plotted using boxplots for time-dependent uncertainty analysis. Effectiveness of interventions was predicted in terms of the ratio of incident HIV cases prevented (Fig. 2A) and the ratio of prevalent HIV cases avoided (Fig. 2B). In other words, the plots above zero illustrate that ratio of HIV cases decreased as compared to the current situation and the plots below zero show that ratio of HIV cases increased. To assess the effect of the level of unsafe sex (UAIC) in PrEP, we compared the number of prevalent HIV cases with scenario 3 and 4-1, 4-2, 4-3 to current situation in Fig. 3. Moreover, as well as the value by plotted using boxplots, the median values of KX and Y_1–4_ in each scenario were shown on Table S2.

### Sensitivity analysis

**Figure 4:**
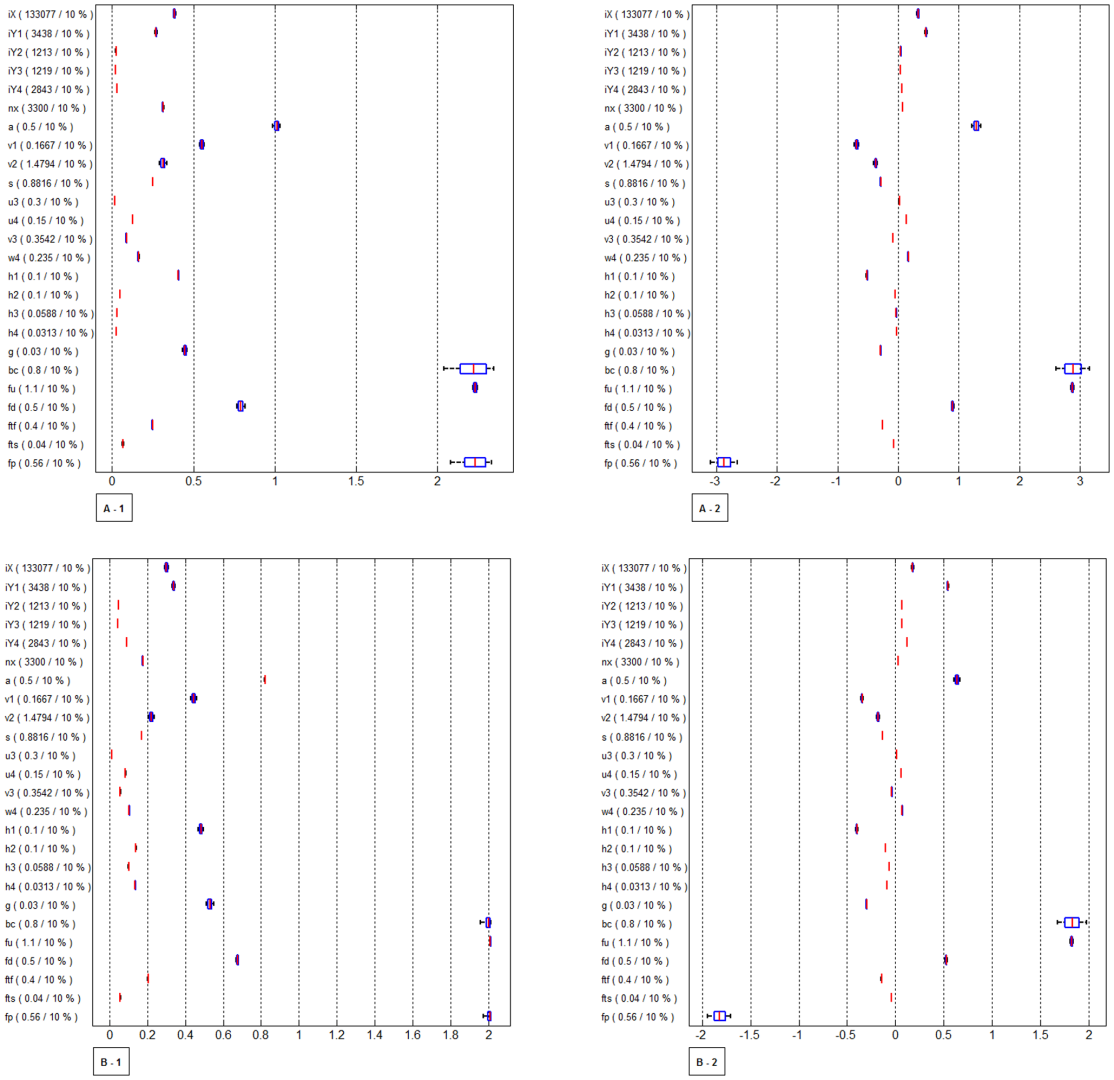
Sensitivity analysis for the number of incident HIV cases (KX) and the number of prevalent HIV cases (Y_1–4_). A-1. Averaged sensitivities in magnitudes of the number of incident HIV cases (KX) per year to each parameter. A-2. Sensitivities of the number of incident HIV cases (KX) per year to each parameter at 10 year. B-1. Averaged sensitivities in magnitudes of the number of prevalent HIV cases (Y_1–4_) per year to each parameter. B-2. Sensitivities of the number of prevalent HIV cases (Y_1–4_) per year to each parameter at 10 year. NOTE: The elasticity of KX with respect to a parameter 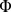 is defined as: 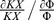. The X-axis of Figures A and B represents the elasticity of incident (KX) and prevalent (Y_1–4_) HIV cases, respectively. See table 1 and S1 for referring the meaning of each abbreviations within Y-axis. Figures 4A-1 and 4B-1 show the average elasticities of outcomes over 40 years and Figures 4A-2 and 4B-2 show the elasticities of outcomes at the time point of 10 years. The boxplot contains the median value (horizontal red line), and extends from the first to the third quartile when simple random sampling with uniform distributions between ±10% of baseline of all parameters are used. Whisker bars are extended to minimum and maximum.



The presence of parameters (including the initial values), none of which are known with confidence, makes a comparison of the various intervention strategies difficult. To observe the effect of uncertainty in parameters on outcomes, sensitivity analysis was performed. A picture of the elasticity of outcomes with respect to the parameters is given in Fig. 4. To generate Fig. 4, 1000 sets of parameter values were randomly sampled from uniform distributions between the limits of ±10% of baseline. Fig. 4-A and 4-B show boxplots of the elasticities of the number of incident (KX) and prevalent (Y_1–4_) HIV cases to each parameter. A boxplot is a graphical representation of the quartiles of a data set in which the box contains the median value of the data, and extends from the first to the third quartile. The elasticity of KX with respect to a parameter 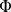 is defined as:

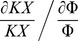
.

## Results

### Current situation

We defined the ‘current situation’ in South Korea as one where an epidemic could be expected to occur in the future if new prevention measures are not undertaken. Our predictions are based on a mathematical simulation, which suggested that the number of incident HIV cases would increase from 3262.5 to 8469.1 in the first 10 years, but start to fall to 5944.5 in the next 15 years, and fall to 2610.4 in 40 years. The simulation also indicated that the number of prevalent HIV cases would increase from 8713 to 60465.5 in the first 15 years, fall to 58319.6 in the next 20 years, and fall to 37651.6 in 40 years. According to our predictions, the HIV incidence rate among the MSM population in South Korea will range between 0.01% and 0.03% in the first 5 years, and the HIV prevalence rate among the MSM population in South Korea will range between 0.03% and 0.11% in the same time frame.

### Early ART (Scenario 1)

If 95% of HIV-diagnosed MSM take ART within 1 year of diagnosis, the number of incident HIV cases would be reduced by 13% compared to the current situation in the first 10 years, but ultimately it would be similar to the current situation at 40 years (Fig. 2A-1). Similarly, the number of prevalent HIV cases would be reduced only by 3% compared to the current situation in the first 20 years (Fig. 2B-1).

### Early diagnosis (Scenario 2)

If HIV-infected individuals were diagnosed with HIV infection within the first year of infection (scenario 2), the number of incident HIV cases would be reduced by 71% in the first 10 years. It would be reduced by 23% in the next 20 years, and the number of incident HIV cases would be similar to the current figure at 40 years (Fig. 2A-2, Table S2). With early diagnosis, the number of prevalent HIV cases would be reduced by 61% in the first 15 years and 40% in the next 25 years, and the number of prevalent HIV cases would be similar to the current figure at 40 years (Fig. 2B-2, Table S2). Despite the uncertainty of the parameters, the preventive effects of early diagnosis were somehow certain before 20 years because most of the boxplots were above zero. The reliability of the preventive effects of an early diagnosis seemed to decrease after 20 years.

### PrEP (Scenarios 3) and the effect of unsafe sexual behavior (Scenario 4)

Based on previous studies in MSM, the effectiveness of PrEP was deemed to be 44% in the model simulations [10]. We also modeled the effectiveness of PrEP based on changes in sexual risk. When there was no increase in unsafe sexual behavior (Scenario 3), the number of incident HIV cases would be reduced by 75% in the first 5 years and 77% in the next 10 years. It would be reduced by 66% in 15 years, 28% in 25 years, and 20% in 40 years as compared to the current situation (Fig. 2A-3, Table S2). Similarly, the number of prevalent HIV cases would be reduced by 69% in the first 10 years and 37% at 40 years as compared to the current situation (Fig. 2B-3, Table S2). Similar to early diagnosis, the preventive effects of PrEP were certain before 20 years because most boxplots were above zero. However, the reliability of preventive effects of an early diagnosis seemed to decrease after 20 years.

We then evaluated PrEP when its effectiveness was reduced by an increase in unsafe sex practices (Fig. 3, Table S2). When the effectiveness of PrEP was reduced by a 10% increase in unsafe sex behavior, the number of incident HIV cases would be reduced by 69% in the first 5 years as compared to the current situation and by 26% in the next 20 years as compared to the current situation (Fig. 3A, Table S2). Similarly, the number of prevalent HIV cases would be reduced by 63% in the first 10 years and by 20% in 40 years as compared to the current situation (Fig. 3B, Table S2). When the effectiveness of PrEP was reduced by a 20% increase in unsafe sexual practices, the number of incident HIV cases would be reduced by 62% in 10 years but was similar to the current situation in 40 years (Fig. 3A, Table S2). The number of prevalent HIV cases would be reduced by 56% in the first 10 years but only by 10% in 40 years as compared to the current situation (Fig. 3B, Table S2). When the effectiveness of PrEP was decreased by a 30% increase in unsafe sex behavior, the number of incident HIV cases would be reduced by 52% in 10 years, but it was similar to the current situation in 40 years (Fig. 3A, Table S2). The number of prevalent HIV cases would be reduced by 49% in the first 10 years but only by 4% in 40 years as compared to the current situation (Fig. 3B, Table S2). Accordingly, our scenario showed that PrEP would be more effective in reducing HIV transmission over the next 15 years compared to the current situation. Although the potential reduction could be counterbalanced by unsafe sex, this would not be sufficient to negate the improvement completely.

### Combination interventions (Scenario 5)

If the interventions were combined to evaluate the maximal effect, the incidence of HIV would decrease sharply from 1827 to 408.4 cases in the first 5 years and to 166.8 cases in 40 years. The incidence would be reduced by 94.4% in the first 5 years and 93.7% in 40 years as compared to the current situation (Fig 3A-4, Table S2). Further, the number of prevalent HIV cases would continue to fall from 8712 to 3483.8 through 40 years. Overall, the HIV prevalence would be reduced by 68% in 5 years and 90.8% in 40 years as compared to the current situation (Fig. 3B-4, Table S2).

### Sensitivity analysis

To determine which factors and terms had the greatest effect on estimating HIV prevalence and incidence, we performed a sensitivity analysis. It provides a way to illustrate the effects of parameters on outcomes using derivative values of outcomes with respect to parameters. The derivatives in the sensitivity analysis were approximated by percent changes in outcomes with percent change in the parameter value. Since these sensitivities are time dependent, averaged magnitudes of incident HIV cases over time and at 10 year are displayed in Fig. 4A-1 and Fig. 4A-2, respectively. Computations were repeated for each of the 1000 parameter sets, giving the results of prevalent HIV cases Y_1–4_ per year, displayed in Fig. 4B-1 and Fig. 4B-2. While the averaged sensitivities and at 10 year were not identical, some general statements can be made. In Fig. 4A and Fig. 4B, outcomes are more robust to the changes in parameters if the values are closer the horizontal axis. For example, in the absence of an intervention strategy (i.e. current situation), outcomes were most sensitive to transmission rate (bc) because the values were located furthest from zero. Similarly, when considering an intervention, transmission rate (bc), the level of UAIC (*f_u_*), and the average decrease in infectiousness as a result of PrEP (*f_p_*) had the most crucial effect on outcomes. We observed that some of the parameters, such as treatment cessation rate due to treatment failure (u_3_) and initial values of the number of infected MSM who are diagnosed regardless of treatment (Y_2_, Y_3_,Y_4_), had little effect on HIV prevalence because the values were located quite close to zero with these parameters.

## Discussion

HIV remains one of the world's most challenging public health threats, and if we are going to stem the current epidemic, then we must evaluate every tool possible. One way to evaluate potential prevention measures before their implementation is through the use of mathematical models, as conducted here. Since in South Korea the most common mode of HIV transmission has gradually changed from heterosexual to homosexual contact among men,[1] we evaluated the use of a variety of prevention interventions, as compared to the estimated current situation. Since there are no pertinent data available from the epidemic over the past 10–20 years in South Korea, we only used data available to the current situation where the number of HIV incident cases among MSM would increase from 3262.5 to 7166.3 persons in the first 5 years. This trend is quite reasonable because approximately half of HIV-infected persons are currently ‘late presenters’ and many infected persons are still undiagnosed in South Korea. [15] Even though the HIV incidence rate among MSM in South Korea has varied from 0.01% to 0.03% in the first 5 years of our predictions, it would be still much lower than that of China [29] and Japan.[30] Further, the prevalence of HIV infection among MSM in Japan has been estimated at about 2% based on probability samples,[30] and another Japanese study showed that the HIV prevalence would climb from its current rate of 2.1% to 10.4% without new interventions amongst MSM in Japan.[31] Interestingly, the incidence and prevalence of HIV infection among MSM in South Korea was estimated to be low comparing with neighboring nations. Therefore, we feel that the predicted rapid increase of HIV incident and prevalent cases are reasonable estimates.

Recently, the HIV prevention field has witnessed the first clinical success with oral and topical PrEP in stopping the sexual transmission of HIV-1,[32], [33] and as a result of this successful trial, the US FDA approved the use of PrEP with the combination drug of tenofovir disoproxil fumarate and emtricitabine. Some previous mathematical modeling studies have reported the effect of ART for HIV-infected individuals on HIV incidence,[3]–[5] but there has been no study regarding modeling the effects of PrEP on HIV incidence. Based on the realistic and available data, our mathematical model suggests that PrEP using the combination drug of tenofovir disoproxil fumarate and emtricitabine would decrease HIV incidence among MSM in South Korea. PrEP without increased unsafe sex behavior reduced the HIV incidence by 77% in comparison with current status. Interestingly, even though the usefulness of PrEP could be limited by increased unsafe sex behavior, PrEP still demonstrated a more beneficial effect in HIV incidence than the current situation. The effectiveness of PrEP was still large even when considering an increase in risky behavior by 30%. Interestingly, the use of ART within one year of diagnosis showed very little impact to incidence as compared to the current situation, while diagnosis within one year of infection showed a comparable decrease in incidence as compared to PrEP. In other words, PrEP reduced the HIV incidence by 74% comparing with the use of ART within one year of diagnosis just in 10 years. Moreover, PrEP reduced the HIV incidence by 23% comparing with diagnosis within one year of infection. For our simulations, 95% ART use within one year of diagnosis was selected as the best possible value, but the predictions of HIV incident and prevalent cases through simulation were not much different between 95% or 100% ART use.

In the present study, we did not consider cost-effectiveness of each intervention because we are interested in the change of the incidence or prevalence of HIV after implementation of each intervention. This is especially pertinent, since PrEP is relatively expensive and could be cost prohibitive for public policy implementation.[34] For example, it is unclear from the modeling whether it would be more cost effective to provide PrEP or to increase early diagnosis among MSM. Nevertheless, if the society could bear medical expenses of all possible interventions, the best way to reduce HIV incidence is scenario 5, which includes diagnosing the disease within one year of infection, treating almost all (approximately 99%) the HIV infected persons within one year of diagnosis, taking PrEP and unchanged unsafe sex behavior. In scenario 5, the HIV incidence sharply decreased in 5 years and kept low level for the modeled 40 years of observation. Our mathematical model is optimized for the HIV transmission among MSM in South Korea and cannot reflect all the possible complexities of other epidemics. To overcome these limitations, we utilized the best available epidemiological data as much as possible. Nevertheless, data on some parameters were obtained from previous studies conducted in other countries, including the number of new uninfected MSM each year (nx), the proportion of new infections undiagnosed at seroconversion (a), the treatment cessation rate due to treatment failure (u_3_) and successful treatment (u_4_), the treatment success rate (v_3_), the treatment relapse rate (w_4_), and the rate of AIDS death for infected MSM after successful treatment (h_4_).

Thus, generalization of our results to other populations might be limited and our estimates might also be biased because of including parameter estimates from western countries, as with most mathematical models. These data from other countries could influence the conclusions of our study. Additionally, our derivations of parameters including diagnosis rate (v_1_), treatment uptake rate (v_2_), and rate of AIDS death for infected MSM after treatment failure (h_3_) was not validated, even though they were based on best available epidemiologic data. Considering these limitations and the uncertainties, we chose a simulation approach, permitting many parameters to vary over a range of uncertainty; however, predictions from our models also depended upon the presented derivations and should be interpreted cautiously.

In spite of these limitations, our study has provided a number of unique insights, which should be evaluated in clinical studies to assess real-life viability. Based on these modeling estimates, we feel that such clinical studies should compare the use of PrEP and early diagnosis versus a combined approach among MSM to reduce HIV incidence. Moreover, these clinical efficacy studies should include a component of cost effectiveness to fully evaluate public health feasibility and societal cost.

## Supporting Information

Figure S1 **Transmission equations.**
(TIF)

Table S1 **Variables and parameters for model.**
(DOCX)

Table S2 **Each values for KX and Y_1_ to Y_4_ according to time sequence.** NOTE: See table 1 and S1 for referring the meaning of each abbreviations.
(DOCX)
