# Supplementary figures and images for: Mathematical Modeling of HIV Prevention Measures Including Pre-Exposure Prophylaxis on HIV Incidence in South Korea
Source: PLoS One. 2014 Mar 24;9(3):e90080. doi: 10.1371/journal.pone.0090080 (PMC3963840; doi:10.1371/journal.pone.0090080)

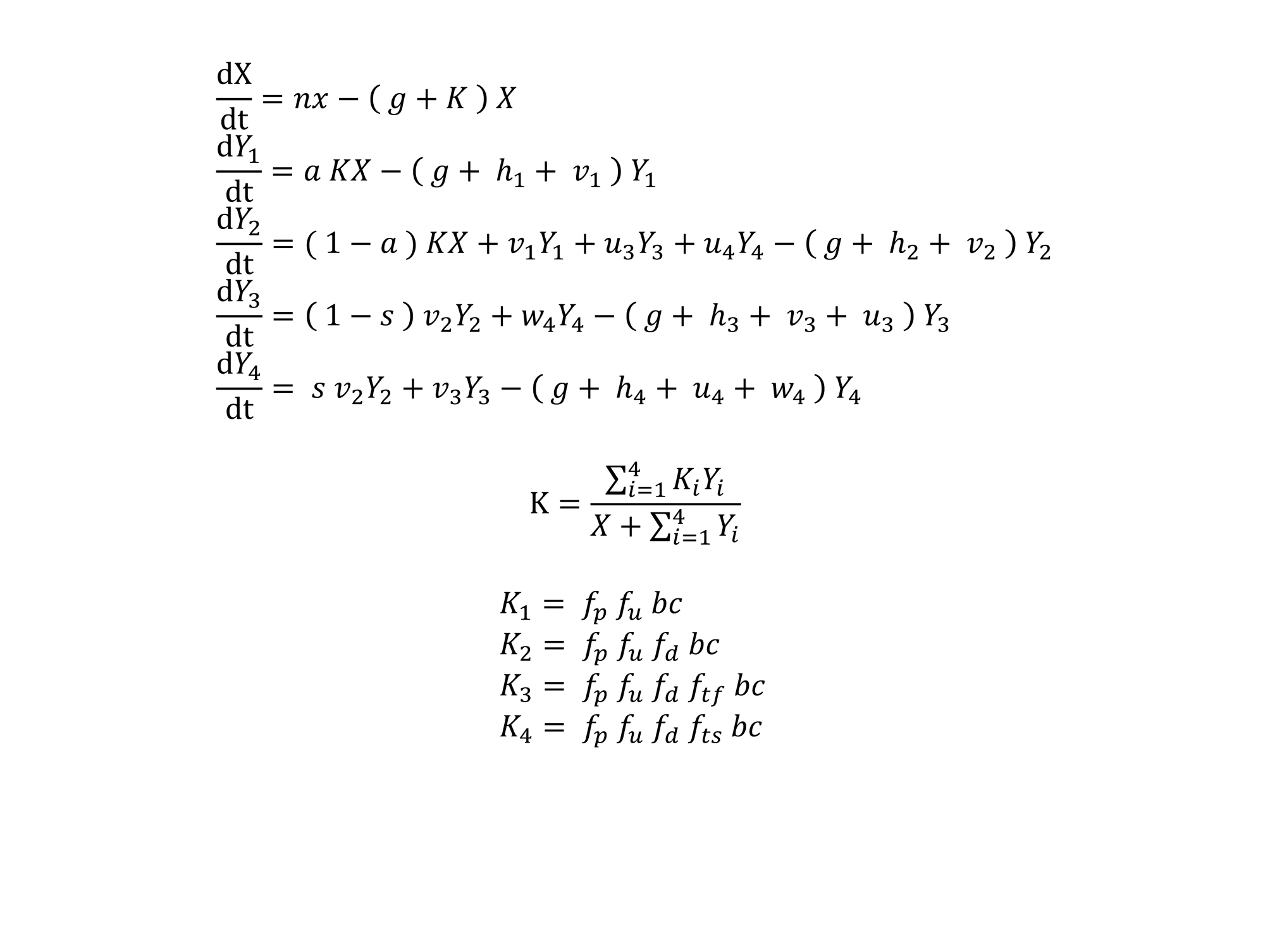

Supplement: Figure S1 — Transmission equations. (TIF) [file pone.0090080.s001.tif]
