# Supplementary material for: Mathematical Modeling of HIV Prevention Measures Including Pre-Exposure Prophylaxis on HIV Incidence in South Korea
Source: PLoS One. 2014 Mar 24;9(3):e90080. doi: 10.1371/journal.pone.0090080 (PMC3963840; doi:10.1371/journal.pone.0090080)
Supplement: Table S1 — Variables and parameters for model. (DOCX) [file pone.0090080.s002.docx]

**TABLE S1.** Variables and parameters for model

| Variables and parameters | Meaning | Explanations |
| --- | --- | --- |
| X | number of uninfected MSM | In 2010, the number of males aged 15-64 years in South Korea were estimated to be 17,602,812.[13] A total of 0.5-1.1% of the male population was derived as the MSM population.[26] Therefore, the MSM population was estimated to be 88,014-193,631. The HIV infection rate among them was derived as 5.5%, according to cited study. [26] Namely, the proportion of HIV uninfected MSM populations among the total MSM populations was derived as 94.5%. Therefore, the number of uninfected persons in the MSM population was estimated to be 83,173-182,981, with 133,077 selected as the median value. |
| Y_1_ | number of undiagnosed, infected MSM | The HIV infection rate among the MSM population was derived as 5.5% , according to a previous study,[26] and the number of infected persons in this population was estimated to be 4841-10650. In 2010, of South Korea, the number of those living with HIV infection was estimated to be 6292. According to a government epidemiological statistical report, the mode of transmission through MSM was estimated to be 45-50% of all those living with HIV. However, the data were limited to persons who answer the question. Only 58.2% of HIV-infected men answered the mode of transmission.[13] Similarly, referring to a previous epidemiology study conducted in South Korea,[2] the number of the mode of transmission through MSM would be higher than estimated because the HIV-infected persons usually conceal their sexual identity secondary to social prejudice. Based on these data and study results and the inherent uncertainty, we concluded that the mode of transmission through MSM was likely higher than the government epidemiology report, and was more likely to be approximately 60-90% of current HIV transmissions. Applying this range, the number of HIV-infected living MSM was estimated to be 3775-5663 persons. Therefore, the number of undiagnosed, infected MSM was estimated to be 1 person at the least and 6875 persons at the most, which was calculated from 3775 to 10650 persons, and 3438 persons was selected as the median value. |
| Y_2_ | number of diagnosed, infected MSM without treatment | Currently, in South Korea, the proportion of HIV infected persons receiving ART was estimated to be 77%. According to most recent data(end of 2011), the number of total survivors was estimated to be 7032.[13] Among these, 4219-6328 persons were derived as HIV-infected MSM. The number of diagnosed, infected MSM persons without treatment was estimated to be 970-1456, with 1213 selected as the median value. |
| Y_3_ | number of infected MSM with treatment failure | If the proportion of HIV infected persons undergoing ART is taken to be 77% of the total number of HIV infected persons, the number of infected MSM undergoing treatment can be estimated to be 3249- 4873. According to a previous study, [14] 73% of HIV-infected persons undergoing ART had viral loads of less than 400 copies/ml in 6 months, and 64.5% of HIV-infected persons receiving ART had viral loads less than 50 copies/ml in 12 months. Based on these figures,[14] the viral suppression rate with ART was derived as 70%, and the failed viral suppression rate was estimated as 30%. The number of infected MSM receiving ART was estimated to be 3249-4873. Among these, the number of infected MSM with treatment failure was estimated to be 975-1462, with 1219 selected as the median value. |
| Y_4_ | number of infected MSM with successful treatment | The number of infected MSM with successful treatment was estimated to be 2274-3411, with 2843 selected as the median value. |
| nx | number of new uninfected MSM each year | The number of new uninfected MSM each year in South Korea has not been reported previously. We derived the value as 3% from previous study, [4] in which there were 90,000 HIV- uninfected MSM and 3,000 new uninfected MSM entering the population annually. Based on these published data, we then estimated the 3% entry into the MSM populations annually. The number(nx) was estimated up to 2100-4500 when the total number of MSM was derived as roughly 70,000-150,000, with 3300 selected as the median value. |
| a | proportion of new infections undiagnosed at seroconversion | Referring to a previous study,[3] the proportion of new undiagnosed infections at seroconversion (a) was estimated to be 0.5. |
| v_1_ | diagnosis rate | We chose to derive our estimates of V_1_ and V_2_ from a previous study conducted in South Korea that showed that more than 70% of newly diagnosed HIV-infected persons had CD4 counts below 350 cells/ mm^3^.[15] From this study, the overall median CD4 cell count at time of diagnosis was 244 cells/mm^3^(IQR 112-379). This allowed to extrapolate the timing of diagnosis on a population level from CD4 counts at diagnosis. Using a European study that estimated times between seroconversion and CD4 cell counts of <500, <350, <200 cells/ mm^3^ were 1.19 years (95% CI,1.12-1.26), 4.19 years (95% CI, 4.09-4.28), and 7.93 years (95% CI, 7.76-8.09).[16] The study also showed that the median CD4 cell count at 1, 2 and 5 years after seroconversion and no treatment was 510 cells/mm^3^ (IQR, 341-721 cells/mm^3^), 460 cells/mm^3^ (IQR, 294-656 cells/mm^3^), and 315 cells/mm^3^ (IQR, 156-528 cells/mm^3^), respectively. Extrapolating from this study, we would expect that our population would be diagnosed within 4.19 years (CD4 cell counts of <350 cells/mm^3^) and 7.93 years (CD4 cell counts of <200 cells/mm^3^). Moreover, referring to a similar study,[17] by 6 years approximately 90% of HIV-infected persons would experience HIV-specific disease (Figure 3.), leading to diagnosis from clinical manifestations. Taken together, we estimated that HIV-infected MSM were diagnosed within 5-7 years of infection. According to our transmission equation (A) which is following: If it takes t years in average to move to the next compartment, then the yearly rate r can be computed by , the 6 years as median value used to calculate it. Therefore, the diagnosis rate (v_1_) was estimated to be 0.1667/year. |
| v_2_ | treatment uptake rate | Generally, ART is started based on a patient’s CD4 cell count, especially the threshold of 350 cells/mm^3^.[18] We then could estimate V_2_ at different CD4 cell counts. As mentioned above, 40% of the newly diagnosed HIV-infected persons had CD4 cell counts <200 mm^3^, which would trigger starting ART within one year from diagnosis, at most. Next, we derived that HIV-infected MSM with CD4 cell counts between 200-349 cells/mm^3^ would be started on ART within one-to-two years from diagnosis, at most. Although no data are available for CD4 counts >350 cells/mm^3^, we divided them into two groups, as follows: CD4 cell counts of 350-500 cells/mm^3^ (20%) and CD4 cell count above 500 cells/mm^3^ (10%). We estimated that the newly diagnosed HIV-infected MSM with a CD4 cell count of 350-500 cells/mm^3^ (20%) would be treated within three years from the time of diagnosis, at most, and the remaining newly diagnosed HIV-infected MSM with a CD4 cell count of >500 cells/mm^3^ w (10%) would be treated within five years from the time of diagnosis, at most. Accordingly, the average duration of treatment uptake from diagnosis was 2.025 years, which was calculated by the following formula: 0.4 as 40% of all HIV-infected persons * 1 year as treatment uptake time+ 0.3 as 30% of all HIV-infected persons * 1.75 years as treatment uptake time+ 0.2 as 20% of all HIV infected persons *3 years as treatment uptake time+ 0.1 as 10% of all HIV-infected persons *5 years as treatment uptake time (In that formula, 1.75 was meant to be 21 months. We picked up the value from ranges between one year and two years). We also derived that the final treatment uptake rate would be 95%, not 100%. According to our transmission equation (B) which is the following: If there z% in t years, then the yearly rate r was computed by , then (v_2_) was estimated to be 1.4794/year. |
| s | proportion of successful treatments | According to a previous study,[14] 70% of diagnosed HIV-infected MSM were successful in suppressing their viral loads in 48 weeks with ART. In the B transmission equation, the proportion of successful treatments (s) was estimated to be 0.8816. |
| u_3_ | treatment cessation rate due to treatment failure | According to an earlier study, [21] the loss to follow-up rate among MSM with failing ART were 29.42/100 person-years. Based on this reported figure, the treatment cessation rate due to treatment failure (u_3_) was estimated to be 0.3/year. |
| u_4_ | treatment cessation rate due to successful treatment | According to a previous study, [21] the loss to follow-up among HIV-infected MSM receiving successful ART was estimated at 16.11/100 person-years. Based on this report, treatment cessation rate due to successful treatment (u_4_) was estimated to be 0.15/year. |
| v_3_ | treatment success rate | According to earlier studies,[22,24] 39-57% of HIV-infected MSM with ART failure had suppressed their viral loads in 96 weeks. In the B transmission equation, the treatment success rate (v_3_) was estimated to be 0.3542/year. |
| w_4_ | treatment relapse rate | According to a previous study,[27] 35-40% of infected persons with suppressed viral loads during ART were shown to have rebounding viral loads in 2 years. In the B transmission equation, the (w_4_) was estimated to be 0.235/year. |
| h_1 &_ h_2_ | rate of AIDS death for undiagnosed & diagnosed MSM | As per an earlier study,[23] the median survival of HIV infected persons following diagnosis regardless of ART, was 10 years. In the A transmission equation, the rate of AIDS death for undiagnosed and diagnosed MSM (h_1_, h_2_) were estimated to be 0.1/year. |
| h_3_ | rate of AIDS death for infected MSM after treatment failure | Based on a previous study,[19] we estimated that the median survival of HIV infected persons following diagnosis with ART failure would be 25 years. Considering that the time from infection to diagnosis was derived as 5-7 years and that the time to diagnosis to taking ART was derived as 2 years, the average duration of survival after ART failure would be 16-18 years. In the A transmission equation, using 17 years as a median, the rate of AIDS death for infected MSM after treatment failure (h_3_) was estimated to be 1/17/year. |
| h_4_ | rate of AIDS death for infected MSM after successful treatment | According to previous studies,[18,25] the median survival of HIV infected persons following diagnosis is 40 years with successful ART. Considering the duration of infection to diagnosis and the duration to receiving ART, the average duration of survival after successful ART would be 31-33 years. In the A equation, using the 32 years as a median, the rate of AIDS death for infected MSM after successful treatment (h_4_) was estimated to be 1/32/year. |
| g | background migration rate for MSM | Since the background migration rate (g) has not been previously described in South Korea, we used a previously published rate from the closest country (i.e. Australia) with a similar epidemic (i.e. MSM).[3] Specifically, we used the published background migration rate for MSM (g) as 1/33.3/year. |
| bc | average probability of HIV transmission occurring with that partner (infectiousness) * average annual number of HIV-infected partners with whom uninfected men have unprotected anal intercourse | As per a previous study, [4] the value used in the current study was derived as 0.8/year. |
| *f_u_* | increased level of UAIC | As per a previous study,[3] we derived a default value of 1 when there was no increase in the level of UAIC. The *f_u_* was determined by the following equation: when there is q% increase in the level of UAIC, $\text{1+0.01*q }$would be estimated. We derived that the implementation of PrEP would result in an increase in the UAIC by 10%, and therefore the final value of *f_u_* was 1.1. |
| *f_d_* | effect of diagnosis on reducing the rate of UAIC between uninfected and diagnosed men | Referring to a previous study,[3] the *f_d_* was calculated from the following equation: $\text{1-0.01*q }$when there is q% reduction on the rate of UAIC around the time of HIV diagnosis. The default value was 0.5 because we derived that the diagnosis would reduce UAIC by 50%. |
| *f_tf_* | average decrease in infectiousness as a result of treatment failure | Since average decrease in infectiousness as a result of treatment failure ( *f_tf_* ) has not been previously published, we derived estimates based on previously published data that partner linked transmission would increase two or three times (HR 2.85) as the baseline viral load increase by 1log copies of HIV RNA/ml. [9] Extrapolating from the study, we estimated that if ART failure also decreased viral loads by 1-2log, then infectiousness would decrease by 1/2 or 1/3. Therefore, average decrease in infectiousness as a result of treatment failure (*f_tf_*) was estimated to be 0.4 (median). |
| *f_ts_* | average decrease in infectiousness as a result of successful treatment | According to a previous study,[9] early ART reduces HIV transmission by 96% to an uninfected partner. Therefore, we derived that successful ART would decrease infectiousness by 0.04. |
| *f_p_* | average decrease in infectiousness as a result of PrEP | *f_p_* was calculated from the following equation: $\text{1-0.44 *0.01*q }$when *f_p_* is given to only q% of applicable ones. According to a previous study, the implementation of PrEP appeared to be associated with a 44% reduction in the incidence of HIV. Therefore, the average decrease in infectiousness as a result of PrEP(*f_p_* ) was estimated to be 0.56. |
| K | HIV infection rate for uninfected MSM | K was calculated from the following transmission equations: $K=\frac{\sum_{i=1}^{4} KY_{i}}{X+\sum_{i=1}^{4} Y_{i}}$ |
| K_1_ | HIV infection rate for infected, undiagnosed MSM | K_1_ was calculated from the following transmission equations: $fpfubc$ |
| K_2_ | HIV infection rate for infected, diagnosed MSM | K_2_ was calculated from the following transmission equations: $fpfufdbc$ |
| K_3_ | HIV infection rate for infected MSM with failing treatment | K_3_ was calculated from the following transmission equations: $fpfufdftf$ |
| K_4_ | HIV infection rate for infected MSM with successful treatment | K_4_ was calculated from the following transmission equations: $fpfufdftsbc$ |

Legend: a, s, v_3_, and v_4_ are calculated by following equation: If there z% in t years, then the yearly rate r was computed by.

v_1_, v_2_, h_1_, h_2_,h_3_, and h_4_ are calculated by following equation: If it takes t years in average to move to the next compartment, then the yearly rate r was computed by . K,K_1_,K_2_,K_3_,K_4_ are calculated by transmission equations supplemented by Fig. S1
