# Supplementary material for: Mathematical Modeling of HIV Prevention Measures Including Pre-Exposure Prophylaxis on HIV Incidence in South Korea
Source: PLoS One. 2014 Mar 24;9(3):e90080. doi: 10.1371/journal.pone.0090080 (PMC3963840; doi:10.1371/journal.pone.0090080)
Supplement: Table S2 — Each values for KX and Y1 to Y4 according to time sequence. NOTE: See table 1 and S1 for referring the meaning of each abbreviations. (DOCX) [file pone.0090080.s003.docx]

**TABLE S2.** Each values for KX and Y_1_ to Y_4_ according to time sequence

|  | Initial | 5yrs | 10yrs | 15yrs | 20yrs | 25yrs | 30yrs | 35yrs | 40yrs |
| --- | --- | --- | --- | --- | --- | --- | --- | --- | --- |
| Current status | | | | | | | | | |
| KX | 3262.5 | 7166.3 | 8469 | 5944.5 | 3891.3 | 3008.6 | 2696.6 | 2612.7 | 2610.4 |
| Y_1-4_ | 8713 | 26455.6 | 49528.2 | 60465.5 | 58319.6 | 51898.7 | 45719.2 | 40969.5 | 37651.6 |
| Scenario 1: Early Therapy | | | | | | | | | |
| KX | 3262.5 | 6012.7 | 7428.7 | 5964.1 | 4153 | 3149.7 | 2712.4 | 2549.3 | 2506.2 |
| Y_1-4_ | 8713 | 24076.5 | 43684.4 | 55849.2 | 56945.5 | 52543 | 47116.5 | 42432.1 | 38897.7 |
| Scenario 2: Early Diagnosis | | | | | | | | | |
| KX | 3262.5 | 2153.1 | 2511.2 | 2827 | 3003 | 3038.6 | 2969.4 | 2842.3 | 2697.3 |
| Y_1-4_ | 8713 | 15271.8 | 19705.4 | 24142.8 | 28140 | 31266 | 33304.8 | 34285 | 34404 |
| Scenario 3: PrEP | | | | | | | | | |
| KX | 1827 | 1853.5 | 1952 | 2056.5 | 2134 | 2171.7 | 2171.4 | 2140.8 | 2091.4 |
| Y_1-4_ | 8713 | 12695.6 | 15732.3 | 18217.9 | 20257.7 | 21854.7 | 22999.4 | 23709.2 | 24034.8 |
| Scenario 4-1: PrEP with decrease in efficacy by unsafe sexual behavior | | | | | | | | | |
| KX | 2009.7 | 2273.4 | 2563.7 | 2787.1 | 2890.4 | 2872.3 | 2766.3 | 2619.9 | 2467.9 |
| Y_1-4_ | 8713 | 13928.1 | 18583.7 | 22736.6 | 26154.3 | 28596.6 | 29985 | 30430.7 | 30166.8 |
| Scenario 4-2: PrEP with decrease in efficacy by unsafe sexual behavior | | | | | | | | | |
| KX | 2192.4 | 2759.2 | 3293.6 | 3611.3 | 3628 | 3414.7 | 3110.3 | 2818.6 | 2585.9 |
| Y_1-4_ | 8713 | 15293.1 | 21905.6 | 28003.2 | 32688.8 | 35392.5 | 36185.8 | 35590.5 | 34221.6 |
| Scenario 4-3: PrEP with decrease in efficacy by unsafe sexual behavior | | | | | | | | | |
| KX | 2375.1 | 3316.6 | 4135.4 | 4449.9 | 4202.4 | 3685 | 3184.1 | 2814.2 | 2572.4 |
| Y_1-4_ | 8713 | 16801.1 | 25712.6 | 33859.9 | 39295.8 | 41344 | 40762.1 | 38770.6 | 36333.1 |
| Scenario 5: All interventions combined | | | | | | | | | |
| KX | 1827 | 408.4 | 351 | 309.2 | 272.9 | 241 | 213 | 188.4 | 166.8 |
| Y_1-4_ | 8713 | 8688.1 | 7597.1 | 6647.4 | 5825.7 | 5113 | 4493.7 | 3954.4 | 3483.8 |

See table 1 and S1 for referring the meaning of each abbreviations
